# Components of Height and Blood Pressure among Ellisras Rural Children: Ellisras Longitudinal Study

**DOI:** 10.3390/ijerph13090856

**Published:** 2016-08-27

**Authors:** Nthai Ramoshaba, Kotsedi Monyeki, Leon Hay

**Affiliations:** 1 Department of Physiology and Environmental Health, University of Limpopo, Polokwane 0700, South Africa; pro.elfas@gmail.com; 2 Department of Physiology, Sefako Makgatho Health Science University, Pretoria 0001, South Africa; leon.hay@smu.ac.za

**Keywords:** blood pressure, cardiovascular disease, hypertension, components of height, rural children, South Africa

## Abstract

To date, there has been no study done investigating the relationship between the components of height and blood pressure (BP) in rural South African children. Therefore, the aim of this study was to investigate the relationship between height, sitting height (SH), leg length (LL), and SH-to-height ratio (SH/H) with BP in Ellisras rural children. All children underwent anthropometric and BP measurements using standard procedure. Linear regression was used to assess the relationship between height, SH, LL, SH/H, and BP. The regression showed a positive significant (*p* < 0.001) association between systolic BP (SBP) with height and SH (β ranged from 0.127 to 0.134 and 95% CI ranged from 0.082 to 0.415). Diastolic BP (DBP) also showed a positive significant (*p* < 0.001) association with height and SH (β ranged from 0.080 to 0.088 and 95% CI ranged from 0.042 to 0.259). After having been adjusted for age, gender, body mass index, and waist circumference, DBP showed a positive significant (*p* < 0.05) association with height. There was a positive significant association between DBP and SBP together with the components of height amongst Ellisras rural children.

## 1. Introduction

Cardiovascular diseases (CVD) are now major public health problems in Africa. From prospective studies, it is also known that risk factors for CVD start early in life and increase morbidity and mortality in sub-Saharan African adults [1,2]. Economic development in South Africa has led to lifestyle changes that contribute to a high prevalence of high blood pressure (BP) and type 2 diabetes [3,4]. In clinical practice, growth proportion may be used to detect growth abnormalities which may lead to CVD in children over time. 

Growth proportion is mostly represented by components of height, sitting height (SH), leg length (LL) and SH-to-height ratio (SH/H). Consequently, high SH/H indicates relatively shorter legs for total height and low SH/H indicates relatively long legs for total height [5]. Many studies reported that BP is closely associated with growth proportion in adults from both developed and developing countries [6,7].

It is well known that greater height is correlated with higher BP in children and adolescents in developed countries [8,9]. Recently, Regnault et al. [10] also reported that SH and height were significantly associated with BP in childhood in developed countries. In children and adolescents, the normal range of BP is determined by body size and age. BP standards that are based on sex, age, and height provide a more precise classification of BP according to body size [11,12]. The prevalence of hypertension in Ellisras children ranges from 1.0% to 11.4%, while overweight prevalence ranges from 0.6% to 4.6% [13]. Waist circumference, body mass index, triceps and subscapular skinfolds showed significant correlation with BP among Ellisras rural children [14]. However, to date, no studies have been done to assess whether or not height, LL, SH, and SH/H can predict high BP in rural South African children. Therefore, the purpose of this study was (1) to compare the SH and SH/H of Ellisras children with reference data (National Health and Nutrition Examination Survey III); (2) to investigate the relationship between height, SH, LL, and SH/H with BP among Ellisras children. We hypothesize that there will be a significant association between components of height and BP among Ellisras rural children. Sitting height, rather than leg length, may explain the positive association of height and BP in childhood. Furthermore, adequate perfusion of a child’s brain and BP at heart level must exceed hydrostatic pressure induced by the vertical distance between the heart and the head, of which the components of height may best predict BP at heart level [10].

## 2. Materials and Methods

### 2.1. Sample

Details of the Ellisras Longitudinal Study (ELS) areas have been reported elsewhere [14]. ELS initially followed a cluster sampling method. Briefly, the study was undertaken at 22 schools (10 preschools and 12 primary schools) randomly selected from 68 schools within the Ellisras area. Birth records were obtained from the principals of each school. Out of 2238 subjects who were sampled at baseline (November 1996), 35 subjects were excluded because their ages were not verified against health clinic records. 

Each of the 22 selected schools was assigned a grade. It was done with the expectation that most of the children in a particular age category (3–10 years) would be found in that grade. In May 1999, Medical Students from Vrije University, Amsterdam, The Netherlands included for the first time the systolic BP (SBP) and diastolic BP (DBP) parameters in the ongoing anthropometric measurements of the ELS. A total number of 1961 subjects (1029 boys and 932 girls), aged 5–12 years, completed all the anthropometric and BP measurements and were considered for analysis. 

The Ethics Committee of the then University of the North, now known as University of Limpopo, granted ethical approval prior to the survey (Project Identification ID: MREC/P/204/2013: IR), and the parents or guardians were provided with written informed consent.

### 2.2. Anthropometry

The children were anthropometrically measured using the method of the International Society for the Advancement of Kinathropometry (ISAK) [15]. A Martin anthropometer was used to measure height to the nearest 0.1 cm. Sitting height (SH) was measured by bringing the horizontal bar of the Martin anthropometer into the most superior midline of the head while the child was sitting in an erect position on a flat stool or box. Leg length (LL, the height from the floor to the landmark trochanterion) was measured with the subject’s feet standing together and the lateral aspect of their right leg against the box. The base of the caliper was placed flush on top of the box, and the caliper was oriented vertically upwards with the moving arm positioned at the marked trochanterion site [15]. 

### 2.3. Blood Pressure

Using an electronic Micronta monitoring kit, at least three BP readings of systolic blood pressure (SBP) and diastolic blood pressure (DBP) were taken after an interval of five minutes. After that, the child was seated quietly for 5 min, with his or her back supported, feet on the floor, and right arm supported, cubital fossa at heart level [16,17]. The bladder of the device contains an electronic infrasonic transducer that monitors the BP and pulse rate, displaying these concurrently on the screen. This versatile instrument has been designed and validated for research and clinical purposes [18]. In a pilot study conducted before the survey, a high correlation (*r* = 0.93) was found between the readings taken with the automated device and those taken with a conventional mercury sphygmomanometer. Pulse pressure (PP) was derived by subtracting SBP from DBP.

### 2.4. Quality Control

All training of anthropometric measurements was done in accordance with the standard procedures of the ISAK [15]. Reliability and validity of anthropometric measurements were reported elsewhere [19]. Briefly, the absolute and relative values for intra- and inter-tester technical error of measurements (% TEM) for height ranged from 0.04–4.16 cm (0.20%–5.01%), which was within the 5.1% acceptable rates as reported by Norton and Olds [20].

### 2.5. Statistical Analysis

Descriptive statistics were presented for stature, SH, LL, SH/H, and BP parameters in the Ellisras rural children aged 5 to 12 years. The independent *t*-test was applied to test the significant level (*p* < 0.05) between sexes. SH and SH/H of Ellisras children were compared with NHANES (National Health and Nutrition Examination Survey) III reference population [21]. The Linear regression models were used to assess the relationship between BP parameters and components of height (height, SH, LL, and SH/H) for unadjusted and adjusted to age, gender, body mass index (BMI), and waist circumference (WC). All statistical analyses were performed using the Statistical Package for the Social Sciences (SPSS) version 23 (SPSS, Chicago, IL, USA). The statistical significance was set at *p* < 0.05.

## 3. Results

Table 1 shows descriptive statistics for height, SH, LL, SH/H, PP, and BP of Ellisras rural children aged 5 to 12 years, according to gender and age group. At age 12, girls’ mean heights (145.6 cm) and SH (74.1 cm) were significantly (*p* < 0.05) higher than boys mean heights (142.6 cm) and SH (72.8 cm). Boys and girls did not show significant differences with their mean SH/H throughout the age range.

**Figure 1:**
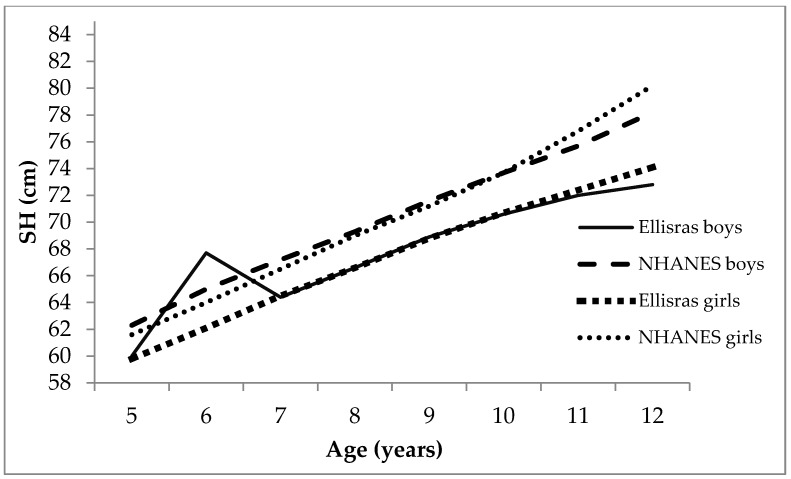
The comparison of SH in Ellisras and National Health and Nutrition Examination Survey (NHANES) children aged 5–12 years.

**Figure 2:**
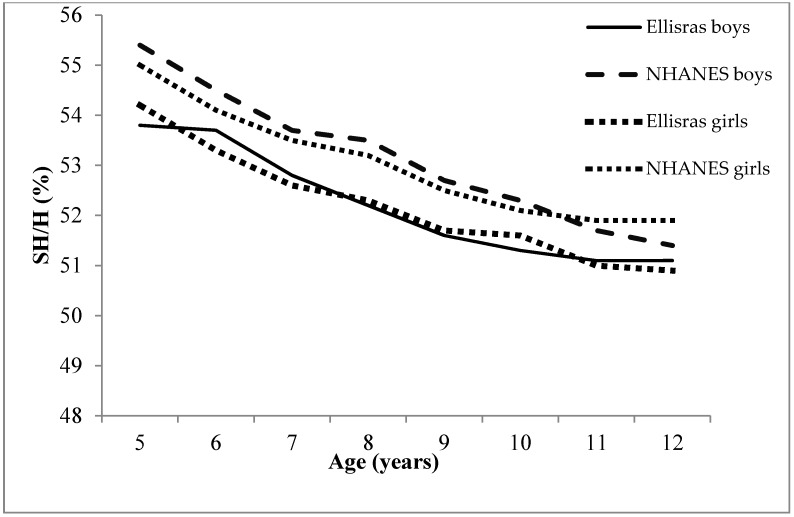
The comparison of SH/H in Ellisras and National Health and Nutrition Examination Survey (NHANES) children aged 5–12 years.

Figure 1 and Figure 2 show the comparison of mean SH and SH/H between Ellisras children and NHANES children aged 5–12 years. There was an increased mean SH with age, and Ellisras rural children had lower mean SH than NHANES children for both genders, except at age 5, which contradicted that of boys (Figure 1). Figure 2 exhibits the decrease in SH/H inversely with age, where mean SH/H amongst Ellisras children were low compared to the NHANES children for both genders. 

The regression analysis showed a positive significant association between SBP with height and SH (*p* < 0.001; β ranged from 0.127 to 0.134 and 95% CI ranged from 0.082 to 0.415). DBP also showed a positive significant association with Height and SH (*p* < 0.001; β ranged from 0.080 to 0.088 and 95% CI ranged from 0.04 to 0.259). After being adjusted for age, gender, BMI, and WC, DBP showed a positive significant (*p* < 0.05) association with height (Table 2).

## 4. Discussion

The purpose of this study was to investigate the relationship between BP with Height, SH, and SH/H among Ellisras rural children. Height and SH of these children were significantly (*p* < 0.05) associated with SBP and DBP for both unadjusted and adjusted with age and gender.

The comparison of NHANES children with Ellisras rural children (Figure 1 and Figure 2) supports the previous study of Monyeki et al. [22], which stated that Ellisras rural children were underweight. Febe et al. [23] reported underweight in four different areas (two from rural and two from urban areas) in South Africa. However, it is possible that nowadays the aforementioned changes and developments in South Africa have resulted in increased body stature for children [24].

Our findings showed that height and SH not SH/H were significantly (*p* < 0.05) associated with both SBP and DBP in the Ellisras rural sample. Thus, it supports Marcato et al. [25] who reported that SH was significantly associated with both SBP and DBP, while SH/H did not show any significant association with SBP and DBP in Brazilian children aged 6 to 13 years. Zhang et al. [26] showed that both SBP and DBP had a stronger association with SH in Chinese children aged 7 to 18 years. 

The possible explanation for the current results could be linked to the hydrostatic column of blood hypotheses formulated by Kahn et al. [27]. Briefly, hydrostatic pressure at a given point increases in proportion to the height of a liquid column because of the increasing weight of fluid exerting the downward force from above. As a result, to ensure adequate perfusion of a child’s brain, BP at heart level must exceed the hydrostatic pressure induced by the vertical distance between the heart and the head, of which the components of height may best predict BP at heart level [10]. The giraffe provides a classic example. A giraffe’s arterial pressure is a consequence of a baroreceptor-regulated mechanism that results in the generation of sufficient hydrostatic pressure to overcome gravitational effects, and to supply the head with blood at a pressure of about 100 mmHg [28]. To generate this pressure, a giraffe’s mean arterial BP is approximately 200 mmHg [29]. 

Reduced fetal and infant growth—which could be related to adult’s height—increases the risk of cardiovascular diseases in adulthood [30]. The recognition of a significant association between components of height and BP in rural South African children in the present study could help to target prevention towards high-risk of CVD in this age group, as evident in other studies [31,32]. Height components are simple, inexpensive tools to identify high-risk of CVD in youth, particularly in the rural South African health sector setting, where resources are limited. Furthermore, the current study indicates that the components of height can be used as a predictor of high BP among children.

In our study, we did not consider the socio-economic status of families of the participants. However, WC and weight have been published elsewhere [14]. Blood samples of ELS subjects were not part of the study. The anthropometric and BP measurements were taken directly; hence, recall or estimation bias will not prevail in our study. In addition, we measured BP during early childhood, demonstrating that proper monitoring should be started from children’s early days from a viewpoint of screening vulnerable individuals [32]. Components of height and BP are more advantageous, due to their applicability and ease of understanding by the general population, particularly in rural South African areas. These indicators could assist in identifying individuals who are at risk of hypertension, which was reported to be central to high mortality and morbidity in Africa [2,4,32].

## 5. Conclusions 

In rural South African children, SH and SH/H are low compared to the reference population (NHANES III). There was a positive significant association between DBP and SBP with the components of height amongst Ellisras rural children. We recommend that further studies be conducted on the relationship between components of height and CVD risk factors overtime in rural South African children.

## Figures and Tables

**Table 1:** Descriptive statistics of components of height and blood pressure parameters of Ellisras children aged 5–12 years.

	Sample Size		Height (cm)		SH (cm)		LL (cm)		SH/H (%)		SBP (mmHg)		DBP (mmHg)		PP (mmHg)	
Age (years)	Boys	Girls	Boys	Girls	Boys	Girls	Boys	Girls	Boys	Girls	Boys	Girls	Boys	Girls	Boys	Girls
			M	M	M	M	M	M	M	M	M	M	M	M	M	M
			(sd)	(sd)	(sd)	(sd)	(sd)	(sd)	(sd)	(sd)	(sd)	(sd)	(sd)	(sd)	(sd)	(sd)
5	53	37	111.6	110.2	60.0	59.8	56.1	56.0	53.8	54.2	100.6	102.3	61.3	59.4	39.3	42.9
			(6.1)	(6.7)	(2.7)	(3.6)	(3.9)	(4.8)	(1.8)	(1.4)	(11.7)	(13.3)	(8.3)	(9.5)	(9.8)	(10.6)
6	64	58	116.9	116.5	67.7	62.1	60.2	60.4	53.7	53.3	102.1	98.4	61.3	59.7	40.8	38.8
			(5.1)	(5.8)	(3.2)	(2.0)	(4.2)	(3.8)	(2.1)	(1.2)	(13.8)	(13.8)	(10.9)	(9.2)	(11.0)	(10.9)
7	97	73	122.2	122.7	64.4	64.5	63.5	64.6	52.8	52.6	96.9	96.3	59.7	57.5	37.1	38.8
			(5.6)	(6.4)	(2.7)	(2.9)	(3.7)	(4.5)	(1.3)	(1.8)	(12.2)	(12.8)	(9.3)	(8.4)	(11.5)	(14.1)
8	117	115	127.7	127.3	66.6	66.6	66.5	67.1	52.2	52.3	97.3	96.4	59.1	59.0	38.2	37.4
			(6.5)	(5.3)	(3.4)	(2.6)	(7.0)	(3.6)	(1.5)	(1.1)	(10.2)	(10.0)	(9.1)	(9.7)	(9.9)	(10.3)
9	184	180	133.5	133.1	68.9	68.8	70.8	70.9	51.6	51.7	99.7	99.0	61.9	61.0	37.9	38.0
			(6.2)	(5.3)	(3.2)	(2.7)	(4.1)	(3.8)	(1.1)	(1.3)	(10.7)	(11.3)	(9.9)	(9.8)	(9.9)	(10.3)
10	231	220	137.7	137.1	70.6	70.7	73.3	73.7	51.3	51.6	99.5	99.7	60.2	59.8	39.3	39.9
			(6.1)	(6.1)	(3.1)	(3.2)	(4.2)	(3.8)	(1.4)	(1.6)	(9.7)	(10.2)	(9.4)	(9.2)	(10.4)	(10.8)
11	187	178	141.0	142.1	72.0	72.4	75.3 *	76.8 *	51.1	51.0	101.6	102.0	62.0	60.9	39.6	41.1
			(7.1)	(6.6)	(3.5)	(3.2)	(4.7)	(4.2)	(1.5)	(1.5)	(10.8)	(11.9)	(9.2)	(9.3)	(9.4)	(11.3)
12	96	71	142.6 *	145.6 *	72.8 *	74.1 *	76.4 *	78.8 *	51.1	50.9	101.9	101.8	60.8	63.1	41.1	38.7
			(6.2)	(7.6)	(3.3)	(3.8)	(4.1)	(4.8)	(1.2)	(1.3)	(9.6)	(10.4)	(8.1)	(8.4)	(10.4)	(9.11)

* *p* < 0.05; M = mean; sd = standard deviation; SH = Sitting height; LL: leg length; SH/H = SH to height ratio; SBP = systolic blood pressure; DBP = diastolic blood pressure; PP = pulse pressure.

**Table 2:** Linear regression coefficients, *p*-value, and 95%confidence intervals for the association between sitting height (SH), SH to height ratio (SH/H), and blood pressures (BP) in Ellisras children age 5–12 years.

	Unadjusted				Adjusted for Age, Gender, BMI, and WC			
	β	*p*-Value	95% CI		β	*p*-Value	95% CI	
**SBP**								
Height	0.127	0.0001	0.082	0.172	0.058	0.2320	−0.037	0.153
SH	0.134	0.0001	0.210	0.415	0148	0.1091	−0.033	0.328
LL	0.187	0.0001	0.119	0.255	0.037	0.6060	−0.102	0.176
SH/H	−0.023	0.3160	−45.141	14.575	17.549	0.3302	−17.806	52.905
**DBP**								
Height	0.080	0.0002	0.042	0.118	0.087	0.0341	0.007	0.168
SH	0.088	0.0001	0.086	0.259	0.108	0.1680	−0.045	0.261
LL	0.121	0.0001	0.064	0.179	0.123	0.0410	0.005	0.241
SH/H	−0.044	0.0540	−49.629	0.407	−11.900	0.436	41.856	18.057
**PP**								
Height	0.047	0.0331	0.004	0.090	−0.029	0.5301	−0.120	0.062
SH	0.140	0.0050	0.041	0.238	0.040	0.6502	−0.135	0.213
LL	0.065	0.0481	0.001	0.130	−0.086	0.2050	−0.220	0.047
SH/H	9.328	0.5182	−18.993	37.649	29.449	0.0490	−4.441	63.338

β = beta, CI = confidence interval; SH = sitting height; LL = leg length; SH/H = SH-to-height ratio; SBP = Systolic blood pressure; DBP = diastolic blood pressure; PP = pulse pressure; BMI = Body mass index; WC = waist circumference.

## Abbreviations

The following abbreviations are used in this manuscript:

AUC	Area under curve
BMI	Body mass index
BP	Blood pressure
CVD	Cardiovascular diseases
DBP	Diastolic blood pressure
ELS	Ellisras Longitudinal Study
ISAK	International Society for the Advancement of Kinanthropometry
LL	Leg length
NHANES III	National Health and Nutrition Examination Survey III
PP	Pulse pressure
SBP	Systolic blood pressure
SH	Sitting height
SH/H	Sitting to height ratio
TEM	Technical error of measurements
WC	Waist circumference
